# Human Papilloma Virus prevalence and type-specific relative contribution in invasive cervical cancer specimens from Italy

**DOI:** 10.1186/1471-2407-10-259

**Published:** 2010-06-04

**Authors:** Luciano Mariani, Núria Monfulleda, Laia Alemany, Enrico Vizza, Ferdinando Marandino, Amina Vocaturo, Maria Benevolo, Beatriz Quirós, Belén Lloveras, Jo Ellen Klaustermeier, Wim Quint, Silvia de Sanjosé, F Xavier Bosch

**Affiliations:** 1 Regina Elena National Cancer Institute, Dept. Gynecologic Oncology, Roma, Italy; 2 Institut Català d'Oncologia (ICO), IDIBELL, Gran Via de L'Hospitalet de Llobregat, Barcelona, Spain; 3 CIBER Epidemiología y Salud Pública (CIBERESP), Barcelona, Spain; 4 Regina Elena National Cancer Institute, Pathology Dept., Roma, Italy; 5 Hospital del Mar, IMAS-IMIM, Barcelona, Spain; 6 DDL Diagnostic Laboratory, Voorburg, The Netherlands

## Abstract

**Background:**

Cervical cancer represents an important global public health problem. It is the 2^nd ^most common cancer among women worldwide. Human Papillomavirus (HPV) infection is now well-established as a necessary cause of invasive cervical cancer (ICC) development. Only a few studies on HPV prevalence and type-specific distribution in ICC have been conducted in Italy.

**Aim:**

To describe the prevalence of HPV and the HPV type-specific distribution in ICC cases identified in Rome, Italy.

**Methods:**

140 paraffin embedded tissue blocks of primary ICC diagnosed between 2001 and 2006 were identified at the Regina Elena Cancer Institute in Rome (Italy). HPV was detected through amplification of HPV DNA using SPF-10 HPV broad-spectrum primers followed by DEIA and then genotyping by LiPA_25 _(version 1).

**Results:**

134 cases were considered suitable for HPV DNA detection after histological evaluation; and overall, 90.3% (121/134) HPV prevalence was detected. 111 cases had a single HPV type, 4 cases had an uncharacterized type (HPVX) and 6 cases had multiple HPV infections. The five most common single HPV types among positive cases were: HPV16 (71/121; 58.7%), HPV18 (12/121; 9.9%), HPV31, HPV45 and HPV58 (5/121; 4.1% each). 2 (1.5%) of the single infections and 2 (1.5%) of the multiple infections contained low risk types. Statistically significant differences in the relative contribution of HPV18 were found when comparing squamous cell carcinomas with adenocarcinomas.

**Conclusions:**

HPV16 and HPV18 accounted for almost 70% of all the HPV positive ICC cases. The study provides baseline information for further evaluation on the impact of recently introduced HPV vaccines in Italy.

## Background

Cervical cancer is a serious public health problem representing the 2^nd ^most common cancer diagnosed among women worldwide. Around 493,000 new cases and 274,000 deaths from this disease were estimated in 2002 [1]. In this study we analyse and present data regarding Human Papillomavirus (HPV) in invasive cervical cancer (ICC) specimens from Italy, a country whose population consist of 25.94 million [2] women at risk of developing ICC (15 y.o. or older). The age-standardized incidence rate of cervical cancer in Italy has been estimated at 8.1 new cases per 100,000 women annually [1]. Current estimates indicate that 3,418 women are diagnosed with ICC every year and 1,186 die from the disease, ranking the 10^th ^most frequent cancer in women, however, the 3^rd ^most frequent cancer in women between 15 and 44 years old [1].

HPV infection is now well-established as necessary but not sufficient cause of ICC development [3-5]. Only a small fraction of women with cervical HPV infection develop ICC, suggesting that other co-factors besides HPV are necessary to cause cancer progression [6]. More than 30 different HPV types are known to infect the cervical epithelium [7,8]. They have been classified into different groups according to their oncogenic potential in ICC: established low-risk types (HPV6, 11, 40, 42, 43, 44, 54, 61, 70, 72, 81 and 89), probable high-risk types (HPV26, 53, 66, 68, 73 and 82) and established high-risk types (HPV16, 18, 31, 33, 35, 39, 45, 51, 52, 56, 58 and 59) [6,9]. Data from an updated meta-analysis conducted by the International Agency for Research on Cancer (IARC) [10,11] confirmed HPV16 as the most common type detected in ICC specimens worldwide, followed by HPV18, together accounting for 70% of the HPVs identified in ICC specimens. After HPV16/18, the next 6 most common HPV types were found to be the same in all world regions (HPV45, 31, 33, 52, 58, and 35), with only slight differences in their relative frequency over continents but, nevertheless, accounting for an additional 20% of cervical cancers worldwide [10,11].

Very few studies on HPV type prevalence and type-specific distribution in ICC have been conducted in Italy [12-17]. Thus, the main aim of this study was not only to present HPV prevalence but also to describe HPV type distribution in ICC specimens from Italy. This study is part of a larger project (Catalan Institute of Oncology-ICO survey) that aims to describe the HPV type-specific distribution in ICC specimens from areas with limited information available using standardized protocols.

## Methods

### Study design and materials

A cross sectional period-prevalence study on archival paraffin embedded ICC specimens was performed. 140 consecutively selected paraffin embedded tissue blocks with ICC from women diagnosed between 2001 and 2006 were identified and retrieved from the Regina Elena Cancer Institute in Rome, Italy. Age at diagnosis and year of diagnosis were collected from medical records.

### Pathology and laboratory procedures: Paraffin blocks processing, histopathological evaluation, HPV DNA detection and typing

Selecting the paraffin blocks and microtome sectioning was performed in Rome, under strict conditions to avoid potential contamination and following a protocol previously developed at ICO. Briefly, at least four paraffin sections were obtained for each block ("sandwich" method). First and last microtome sections were stained with Haematoxylin and Eosin (H&E) for histopathological evaluation. The sections that were cut in-between the two H&E slides were collected in two eppendorf type tubes for HPV DNA testing (one tube for initial testing and the other for back-up). A tissue-free paraffin block was cut after each study block to avoid HPV carry-over from block to block. A new blade was used for each study block, and the microtome was cleaned with Histoclear II, a xylene substitute, and 70% alcohol. Paraffin blocks containing non HPV-related lesions, such as, non HPV-related tumours or non-tumour biopsies were blindly included and analyzed as controls in the process for further quality control of laboratory results.

The pathology classification of the histopathological diagnosis was done at ICO following the consensus criteria established by an expert panel of collaborating pathologists. These criteria were based on the World Health Organization (WHO) classification of uterine cervix tumours [18]. The pathology evaluation included: diagnosis of histological type (ICC: -squamous cell carcinoma, adenocarcinoma, adenosquamous carcinoma, other types-; non-invasive cervical cancer and control tissue specimen); presence of normal mucosa or pre-neoplastic lesions adjacent to ICC (Cervical Intraepithelial Neoplasia - CIN1, 2, or 3; Adenocarcinoma in Situ-AIS); degree of necrosis; tumour infiltration expressed by % (quantity of necrosis/infiltrating component of the tumour in the total of the section), and adequacy of the sample to proceed to HPV testing. A sample was determined to be adequate for HPV analysis if invasive cancer was observed on both H&E stained sections of the study specimen.

DNA isolation was performed by treating the cut sections of paraffin embedded tissues with 250 μl of freshly prepared 0.1% proteinase K solution (5 mM Tris HCl, 0.1 mM EDTA, 0.05% Tween 20). The samples were incubated at 56°C for 18 hours to overnight. Proteinase K was then heat inactivated at 95°C for 10 minutes. Short Polymerase Chain Reaction Fragment - using biotin labelled SPF-10 primers [19,20] was performed using 10 μl of a 1:10 dilution of the crude DNA isolate in a final reaction volume of 50 μl. Presence of HPV DNA, was determined by a probe hybridization technique that contains a mixture of HPV specific probes recognizing at least 54 mucosal HPV genotypes in a microtiter plate format (DNA Enzyme Immuno Assay -DEIA). Briefly, 10 μl of the PCR product was incubated in a hybridization buffer in a streptavidin-coated microtiter plates. The plates were washed and the captured PCR product was denatured using NaOH. Subsequently, digoxigenin (DIG)-labelled HPV-specific probes were added and following incubation, the plates were washed 3 times. Finally, an anti-DIG alkaline phosphatase substrate was added to the wells and after a short incubation time, the optical densities were determined. Optical densities (OD450) were read on a microtiter plate reader (Bio-tek) [19]. For genotyping HPV DNA, 10 μl of the PCR amplimers of the HPV DNA positive samples, as identified by DEIA, were analyzed by Line Probe Assay-LiPA_25 _version 1. (Labo Biomedical Products, Rijswijk, The Netherlands), that can detect 25 different high-risk and low-risk HPV types (6, 11, 16, 18, 31, 33, 34, 35, 39, 40, 42, 43, 44, 45, 51, 52, 53, 54, 56, 58, 59, 66, 68, 70, 74). The sequence variation within the SPF-10 primers allows the recognition of these different HPV genotypes, except for types 68 and 73, as their interprimer regions are identical and cannot be distinguished by this test. Positive hybridization on the strips is visualized as a purple band by means of a precipitating colour substrate on the probe site. Specimens that were identified as HPV DNA positive, but did not hybridize with any of the 28 LiPA probes patterns, were coded as HPV type X (uncharacterized type). SPF-10 PCR, detection and typing were performed at ICO.

### Statistical analysis

Data analysis was performed with the Statistical Package SPSS 14.0. Overall and type-specific HPV prevalence (within the total of samples analyzed) and type-specific relative contribution (within the HPV positive cases) were analyzed and 95% confidence intervals (95% CIs) for proportions were calculated. To estimate the HPV type distribution, single and multiple HPV infections were considered either separately or combined. Stratified analyses of overall HPV and type-specific distributions by age at diagnosis and histological characteristics were performed. Age was recoded as a categorical variable (≤39, 40-49, 50-59, and ≥60 years). Statistical significance for two sided chi-squared and linear trend tests were set at 0.05 levels [21].

### Ethical issues

Specimens were received at the reference laboratory in Barcelona (ICO) under an anonymous manner (without name and/or original medical record number nor histology report). All protocols were approved by local and ICO ethical committees. The study's progress was overseen by an international steering committee specifically created for the supervision and to act as an advisory committee for handing critical ethics as well as scientific issues of the project.

## Results

After histological evaluation, 134 ICC specimens from the original 140 cases were considered suitable for HPV testing; 6 specimens were excluded because they were classified as non-invasive cervical cancers. Mean age of HPV analyzed cases was 52.4 years (Standard Deviation: 14.2; minimum 23 years, maximum value 85 years). Squamous cell carcinoma was the most frequent histological type (83.6%), followed by adenocarcinoma (12.7%) and adenosquamous cell carcinoma (0.8%). Other histopathological types accounted for 3.0%, and were classified as 2 undifferentiated carcinomas, 1 poorly differentiated large cell carcinoma and 1 basal adenoid carcinoma. Microscope slides were systematically reviewed by the pathologists to identify characteristics that could affect detection such as grade of necrosis, tumour infiltration, concomitant lesions or if mucosa was associated. Table 1 describes the different parameters that were evaluated without considering HPV status. The majority of specimens (89.6%) had a low percentage of necrotic cells (grade of necrosis was below 25%). Tumour infiltration under 25% was rarely seen (6.0%). 81.3% of cases had no pre-invasive lesion associated: CIN I was identified in only one HPV positive case, CIN III was observed in 22 cases (16.4%) and AIS lesions were found in 4 cases (3.0%), two of which were concomitant with a CIN III lesion. Almost two thirds of the series had no associated mucosa (66.4%). There were no major differences in HPV positivity by age at diagnosis and histological features evaluated, as shown in Table 1.

**Table 1:** Detection of HPV DNA in ICC specimens from Italy, by age at diagnosis and histopathological information

	HPV analyzed cases (n)	HPV positive cases (n)	**HPV prevalence % (95% CI)**
**Age (years)**			
≤**39**	29	25	86.2 (68.7-96.1)
**40-49**	36	35	97.2 (85.5-99.9)
**50-59**	27	25	92.6 (75.7-99.1)
≥**60**	42	36	85.7 (71.5-94.6)

**Histological type**			
**Squamous cell carcinoma**	112	101	90.2 (83.1-95.0)
**Adenocarcinoma**	17	16	94.1 (71.3-99.8)
**Adenosquamous cell carcinoma**	1	1	100.0 (25.0-100.0)
**Other**	4	3	75.0 (19.4-99.4)

**Degree of Necrosis**			
**<25%**	120	110	91.7 (85.2-95.9)
**25%-50%**	12	11	91.7 (61.5-99.8)
**51%-75%**	2	0	0.0 (0.0-84.2)

**Tumour Infiltration**			
**<10%**	1	1	100.0 (25.0-100.0)
**10%-25%**	7	6	85.7 (42.1-99.6)
**26%-50%**	43	38	88.4 (74.9-96.1)
**51%-75%**	41	39	95.1 (83.5-99.4)
**>75%**	42	37	88.1 (74.4-96.0)

**Mucosa**			
**Squamous mucosa**	15	14	93.3 (68.0-99.8)
**Endocervical mucosa**	10	9	90.0 (55.5-99.7)
**Squamous and endocervical mucosa**	20	18	90.0 (68.3-98.8)
**No mucosa**	89	80	89.9 (81.7-95.3)

CIN III (*)			
**Yes**	22	20	90.9 (70.8-98.9)
**No**	112	101	90.2 (83.1-95.0)

AIS (**)			
**Yes**	4	4	100.0 (39.8-100)
**No**	130	117	90.0 (83.5-94.6)

**Total**	**134**	**121**	**90.3 (84.0-94.7)**

(*) CIN III: Cervical Intraepithelial Neoplasia III - Adjacent to ICC.

(**) AIS: Adenocarcinoma in Situ - Adjacent to ICC.

95% CI: 95% Confidence Interval.

Data on HPV testing and typing is summarized in Table 2. 121 of 134 specimens were HPV DNA positive (90.3%; 95% CI: 84.0%-94.7%). Among the HPV positive cases, 111 were identified as single HPV types (91.7%), 4 were HPVX (3.3%) and 6 harboured multiple HPV types (5.0%). The five most common single types among positive cases were: 71 HPV16 (58.7%), 12 HPV18 (9.9%) and 5 of each HPV31, HPV45 and HPV 58 (4.1%). Multiple infection combinations were: HPV16&18, HPV45&51, HPV31&42, HPV31&52, HPV6&53&66 and HPV53&56&58. In two cases, exclusively low risk types were identified, one HPV42 and one HPV70 as single infections. These were repeated at least twice, and in both occasions showed strong hybridization patterns at the probe sites. HPV6 and HPV42 also were harboured in two multiple infections (HPV6&53&66, HPV31&42).

**Table 2:** Distribution of HPV types in ICC specimens from Italy

	HPV type-specific positive cases (n)	HPV prevalence (95% CI) (*)	Relative HPV type contribution (95% CI) (**)
***Single types***	**111**	**82.2 (75.4-88.8)**	**91.7 (85.3-96.0)**
**HPV16**	71	53.0 (44.2-61.7)	58.7 (49.4-67.5)
**HPV18**	12	9.0 (4.7-15.1)	9.9 (5.2-16.7)
**HPV31**	5	3.7 (1.2-8.5)	4.1 (1.4-9.4)
**HPV45**	5	3.7 (1.2-8.5)	4.1 (1.4-9.4)
**HPV58**	5	3.7 (1.2-8.5)	4.1 (1.4-9.4)
**HPV33**	4	3.0 (0.8-7.5)	3.3 (0.9-8.2)
**HPV35**	2	1.5 (0.2-5.3)	1.7 (0.2-5.8)
**HPV51**	2	1.5 (0.2-5.3)	1.7 (0.2-5.8)
**HPV52**	2	1.5 (0.2-5.3)	1.7 (0.2-5.8)
**HPV42**	1	0.7 (0.0-4.1)	0.8 (0.0-4.5)
**HPV56**	1	0.7 (0.0-4.1)	0.8 (0.0-4.5)
**HPV70**	1	0.7 (0.0-4.1)	0.8 (0.0-4.5)

***Multiple types***	**6**	**4.5 (1.7-9.5)**	**5.0 (1.8-10.5)**
**HPV 16&18**	1	0.7 (0.0-4.1)	0.8 (0.0-4.5)
**HPV 45&51**	1	0.7 (0.0-4.1)	0.8 (0.0-4.5)
**HPV 31&42**	1	0.7 (0.0-4.1)	0.8 (0.0-4.5)
**HPV 31&52**	1	0.7 (0.0-4.1)	0.8 (0.0-4.5)
**HPV 6&53&66**	1	0.7 (0.0-4.1)	0.8 (0.0-4.5)
**HPV 53&56&58**	1	0.7 (0.0-4.1)	0.8 (0.0-4.5)

***HPVX (Unknown type)***	**4**	**3.0 (0.1-5.9)**	**3.3 (0.9-8.2)**

Potential vaccine impact (***)			
**HPV16, 18, 16&18**	84	62.7 (53.9-70.9)	69.4 (60.4-77.5)

**Total HPV positive cases**	**121**	**90.3 (84.0-94.7)**	**100.0**

**Total HPV analyzed cases**	**134**		

(*) HPV prevalence (95% CI), among HPV analyzed cases (n = 134).

(**) HPV type-specific relative contribution (95% CI), among HPV positive cases (n = 121).

(***) Considering a 100% vaccine efficacy (without cross-protection).

95% CI: 95% Confidence Interval.

**Figure 1:**
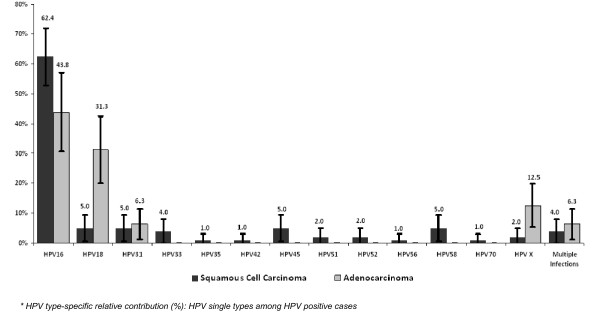
**HPV type distribution among ICC HPV positive cases, regarding histology**.

HPV16 relative contribution was higher in squamous cell carcinoma (62.4%) than in adenocarcinoma (43.8%) with HPV16 as the most frequent type identified in both (p > 0.05). HPV18 was more prevalent in adenocarcinomas (31.3%) than in squamous cell carcinomas (5.0%) (p < 0.05). HPV31, HPV45 and HPV58 single infections were as frequent as HPV18 in squamous cell carcinoma (5.0%) (Figure 1).

The relative contribution of HPV16 was inversely related to increasing age. Nearly 72% HPV16 single types were identified within the youngest age group (<40 years), 57% in the 40-49 years group and 54% in women older than 49 years; although these differences were not statistically significant, p-trend test >0.05.

## Discussion

In the present study a high proportion (90.3%) of the ICC specimens analyzed was HPV DNA positive. HPV16 and HPV18 together accounted for 69.4% of the HPV positive cases. Types HPV31, HPV45, HPV58 and HPV33 also were identified with no major distinction between them in terms of relative importance. Collectively, these types accounted for an additional 15.6% of single infections.

Several meta-analyses on HPV detection among ICC have been published by IARC [10,11] presenting an overall HPV prevalence by geographical area ranging from 79.3% in Asia to 88.1% in North America. In published data updated in 2007 [11], the HPV positivity increased to 87% (ranging from 86% to 94% by region). These variations or the increase of the HPV positivity could be explained by the differences on the methodologies used to determine HPV DNA positivity, histopathologic quality of the samples and the type of specimens analyzed (biopsies, surgical specimens, fresh/frozen tissue samples). The overall HPV positivity in our study was even slightly higher given that a highly sensitive PCR technology was used [19] along with a prior examination of the tissue sections by pathologists to ensure the quality of the specimens before continuing onto testing.

3,607 women with incident and histological confirmed cervical cancer recruited in 25 countries were used for a pooled analysis of data in an international survey of cervical cancer HPV types and a multicenter case-control study, both co-coordinated by the IARC [22]. The study published the two most common HPV types as follows: 57.4% prevalence for HPV16 (from 52% in Asia to 58% in Europe) and 16.6% prevalence for HPV18 (from 13% in South and Central America to 22% in North America). In our study HPV16 also was the most common type in ICC, with a prevalence of around 53%, in agreement with previous reports. Although HPV18 was the second most frequent type, its prevalence of approximately 9.0% in Italy was considerably lower compared with previous reports but still in agreement with previous studies conducted in Italy (Table 3) [12-17]. Our data were in agreement regarding HPV16 prevalence with other studies conducted in Italy. Although based on small numbers, in our study we observed HPV58 to be as prevalent as HPV45, which was consistent with previous observations [10,11]. Moreover, when compared with data from the WHO/ICO HPV information centre for ICC (Table 4), in neighbouring regions and countries, HPV16 and 18 almost were first and second HPV types in frequency position, followed by HPV31, 33, 45, 51 and 52 with slightly prevalence variation [23].

**Table 3:** Comparison of HPV prevalence in ICC specimens among studies conducted in Italy

Author	Year	Tecnique used	Samples analized	HPV + cases (%)	HPV16	HPV18	HPV31	HPV33	HPV45	HPV58	HPVx	Other	M.I.
**Gioele G. Garzetti *et al. ***[13]	1998	PCR primers for HPV6, 11, 16, 18, 31, 33, 34, 35, 42, 51, 56 & 58	**32**	22 (68.8%)	**16** **(50.0%)**	**4** **(12.5%)**	**2** **(6.3%)**	**0** **(0.0%)**	-	**0** **(0.0%)**	**1** **(3.1%)**	**0** **(0.0%)**	**1** **(3.1%)**
**Ciotti *et al. ***[14]	2005	MY09/11, GP5+/6+	**102**	92 (90.2%)	**59** **(57.8%)**	**8** **(7.8%)**	**6** **(5.9%)**	**10** **(9.8%)**	**3** **(2.9%)**	**4** **(3.9%)**	**0** **(0.0%)**	**14** **(13.7%)**	**12** **(11.8%)**
**Del mistro *et al. ***[15]	2006	GP5+/6+	**48**	44 (91.7%)	**32** **(66.7%)**	**4** **(8.3%)**	**1** **(2.1%)**	**4** **(8.3%)**	**2** **(4.2%)**	**2** **(4.2%)**	**0** **(0.0%)**	**4** **(8.3%)**	**5** **(10.4%)**
**Tornesello *et al. ***[16]	2006	MY09/11 & GP5+/6+	**65**	53 (81.5%)	**39** **(60.0%)**	**4** **(6.2%)**	**3** **(4.6%)**	**4** **(6.2%)**	**0** **(0.0%)**	**0** **(0.0%)**	**0** **(0.0%)**	**4** **(6.2%)**	**1** **(1.5%)**
**Gargiulo *et al. ***[17]	2007	Linear array	**31**	29 (93.5%)	**19** **(61.3%)**	**4** **(12.9%)**	**3** **(9.7%)**	**4** **(12.9%)**	**4** **(12.9%)**	**1** **(3.2%)**	**0** **(0.0%)**	**28** **(90.3%)**	**14** **(45.2%)**
**Present study**	2008	SPF10/DEIA/LIPA25	**134**	121 (90.3%)	**72** **(53.7%)**	**13** **(9.7%)**	**7** **(5.2%)**	**4** **(3.0%)**	**6** **(4.5%)**	**6** **(4.5%)**	**4** **(3.0%)**	**17** **(12.7%)**	**6** **(4.5%)**

**AVERAGE**			**412**	**361** **(87.6%)**	**237** **(57.5%)**	**37** **(9.0%)**	**22** **(5.3%)**	**26** **(6.3%)**	**15** **(3.6%)**	**13** **(3.2%)**	**5** **(1.2%)**	**67** **(16.3%)**	**39** **(9.5%)**

**M.I.: Multiple infections**. A multiple infection is counted once on every type involved. In example: HPV16&74 is counted as n = 1 in HPV16 and n = 1 in HPV74. The sum of the total % may exceed 100%.

**Gioele G. Garzetti et al**.: **Histology**: 32 patients with FIGO Stage I and IIA SCC who had undergone primary radical hysterectomy and bilateral oophorectomy. Patients with no SCC histotype were excluded. **M.I**.: HPV16&31.

**Ciotti et al**.: **Histology**: 102 SCC. **M.I**.: n = 4 HPV31&54; n = 3 HPV16&33; n = 3 HPV16&18; n = 1 HPV33&54; n = 1 HPV16&53. **Other**: n = 8 single infections (n = 3 HPV56; n = 2 HPV52, n = 1 HPV51, n = 1 HPV35, n = 1 HPV73) and n = 6 in M.I. (n = 4 HPV31&54, n = 1 HPV33&54, n = 1 HPV16&53).

**Del Mistro et al**.: **Histology**: 48 SCC (43 SCC and 5 Adenocarcinomas)**. M.I**.: n = 5 (n = 3 HPV16&18; n = 1 HPV16&31; n = 1 HPV56&70).**Other**: n = 2 in single infections (n = 1 HPV35; n = 1 HPV52) and n = 2 in M.I: (n = 1 HPV56; n = 1 HPV70).

**Tornesello et al**.: 65 SCC. **M.I**.: n = 1 HPV16&73. **Other**: n = 3 single infections (n = 1 HPV35, n = 1 HPV62, n = 1 HPV82) and n = 1 M.I (HPV16&73).

**Gargiulo et al**.: **Histology**: 31 cervical cancer samples analyzed with non histological sub classification specified. **M.I**.: n = 14, already counted in Table 2 of the article. They do not specify all the M.I as some were up to more the 5 HPV types. Only the most prevalent ones are described. **Other**: n = 5 HPV52; n = 4 HPV61; n = 3 HPV70; n = 3 HPV84; n = 2 HPV51; n = 2 HPV62; n = 1 HPV35; n = 1 HPV39; n = 1 HPV56; n = 1 HPV53; n = 1 HPV55; n = 1 HPV66; n = 1 HPV67; n = 1 HPV81; n = 1 HPV89.

**Present study**: **Histology**: 134 samples (122 SCC, 17 Adenocarcinomas, 1 adenosquamous and 4 other histological types). **M.I**.: n = 6 (n = 1 HPV16&18; n = 1 HPV45&51; n = 1 HPV31&42; n = 1 HPV31&52; n = 1 HPV6&53&66; n = 1 HPV53&56&58). **Other**: n = 9 in single infections (n = 2 HPV35; n = 2 HPV51; n = 2 HPV52; n = 1 HPV42; n = 1 HPV56; n = 1 HPV70) and n = 8 in M.I (n = 2 HPV53; n = 1 HPV51; n = 1 HPV52; n = 1 HPV42; n = 1 HPV6; n = 1 HPV66; n = 1 HPV56).

**Table 4:** The five most common HPV types (HPV type-specific prevalence) in ICC specimens for any histology: Worldwide, Europe, Southern Europe region and neighbourhood European countries

HPV Position	**Italy**^**a**^	**World**^**b**^	**Europe**^**b**^	**Southern Europe**^**b**^	**Austria**^**b**^	**Croatia**^**b**^	**France**^**b**^	**Germany**^**b**^	**Greece**^**b**^	**Spain**^**b**^
**1**^**st**^	**HPV16** **(53.7%)**	HPV16 (54.4%)	HPV16 (57.7%)	HPV16 (48.5%)	HPV16 (70.0%)	HPV16 (45.0%)	HPV16 (60.7%)	HPV16 (58.0%)	HPV16 (22.2%)	HPV16 (51.0%)
**2**^**nd**^	**HPV18** **(9.7%)**	HPV18 (16.5%)	HPV18 (16.8%)	HPV18 (13.6%)	HPV33 (21.5%)	HPV18 (36.9%)	HPV18 (14.9%)	HPV18 (18.8%)	HPV18 (19.1%)	HPV31 (5.3%)
**3**^**rd**^	**HPV31** **(5.2%)**	HPV58 (5.1%)	HPV33 (4.3%)	HPV31 (6.5%)	HPV18 (8.5%)	HPV51 (17.6%)	HPV31 (3.8%)	HPV52/68 (5.6%)	HPV6 (3.8%)	HPV18 (4.9%)
**4**^**th**^	**HPV45/58** **(4.5%)**	HPV33 (4.7%)	HPV31 (4.1%)	HPV33 (4.9%)	HPV45 (4.5%)	HPV31/33 (11.7%)	HPV33/68 (3.3%)	HPV31 (2.9%)	HPV51 (2.9%)	HPV45 (4.1%)
**5**^**th**^	**HPV33** **(3.0%)**	HPV45 (4.4%)	HPV45 (3.5%)	HPV45 (3.3%)	HPV31 (4.0%)	HPV45 (2.7%)	HPV45 (2.9%)	HPV35 (1.4%)	HPV33 (2.8%)	HPV33 (3.0%)

a) Data for Italy: extracted from the present study.

b) Source of information: WHO/ICO Information Centre on Human Papillomavirus (HPV) and Cervical Cancer http://www.who.int/hpvcentre/statistics/en/ (ref.[23]); Selection of regions: World, Europe, Southern Europe (Albania, Andorra, Bosnia & Herzegovina, Croatia, Greece, Italy, Macedonia, TFYR, Malta, Montenegro, Portugal, San Marino, Serbia, Slovenia, Spain), and neighbourhood European countries; No data available for: Bosnia & Herzegovina, Montenegro, Slovenia and Switzerland.

Methods for counting HPV type-specific prevalence: HPV single and multiple infections counted once in each HPV type; prevalence: HPV positive cases for a specific HPV type among HPV analyzed cases.

The association between HPV type and histological type has been described in a large number of studies [10,11]. *Tenti et al. *reported a slightly higher prevalence of HPV18 (29.7%) than HPV16 (28.3%) in a study conducted in Italy on 138 adenocarcinomas of the cervix [12]. Similarly, in the present study HPV16 was shown to be more common in squamous cell carcinomas whereas HPV18 was more common in adenocarcinomas.

Even though a high-sensitive HPV DNA PCR based technology was used for detection and HPV typing (SPF-10-LiPA_25_), our study is not without some limitations. Sample collection was available from only one hospital in Italy (the Regina Elena National Cancer Institute of Rome). Although the number of samples analyzed is limited and obviously not representative of the whole country, they were originated from a systematic collection at a national cancer reference centre providing a potential good representation of all cases in the country.

## Conclusions

Our data is in good agreement with previously published information and indicate that a HPV vaccine targeting HPV types 16 and 18 could potentially prevent 70% of HPV positive cervical cancers. The earlier age at diagnosis of most HPV 16 related cancers may have implications in future strategies of prevention in adult women.

## Competing interests

Partial support has been obtained from Spanish public grants from the Instituto de Salud Carlos III (grants FIS PI030240, FIS PI061246, RCESP C03/09, RTICESP C03/10, RTIC RD06/0020/0095 and CIBERESP), from the Agència de Gestió d' Ajuts Universitaris i de Recerca (AGAUR 2005SGR 00695), the Marató de TV3 Foundation (051530), and from GlaxoSmithKline Biologicals, Sanofi Pasteur MSD & Merck & Co, Inc., who had no role in the data collection, analysis, or interpretation of the results.

## Authors' contributions

LM, EV, FM, AV and MB were responsible for the specimens' selection. BLL was responsible for the pathology evaluation. JK and WQ were responsible for the HPV DNA detection and quality control analysis. BQ, LA, and NM were responsible for the data analysis. NM, LA and SdS contributed to the writing of the manuscript and LM, NM and LA were responsible for the preparation of the manuscript for submission. XFB and SdS planned the international collaborative project on HPV in invasive cervical cancer (ICO survey-RIS HPV TT). All authors read and approved the final manuscript.

## Pre-publication history

The pre-publication history for this paper can be accessed here:

http://www.biomedcentral.com/1471-2407/10/259/prepub
